# Exploring the Role of the Pulvinar Nucleus of the Thalamus in Occipital Lobe Epilepsy: A Case Report

**DOI:** 10.7759/cureus.52534

**Published:** 2024-01-18

**Authors:** Hael Abdulrazeq, Anna R Kimata, Andrew Blum, Athar N Malik, Wael F Asaad

**Affiliations:** 1 Department of Neurosurgery, The Warren Alpert Medical School of Brown University, Providence, USA; 2 Department of Neurology, The Warren Alpert Medical School of Brown University, Providence, USA; 3 Department of Neuroscience, The Warren Alpert Medical School of Brown University, Providence, USA

**Keywords:** intracranial electroencephalography, epilepsy surgery, thalamus, pulvinar nucleus, neuromodulation

## Abstract

Understanding the role of the pulvinar nucleus may be critical for guiding circuit-targeted neurosurgical intervention in some patients. In this report, a 33-year-old female presented with focal onset occipital epilepsy with secondary generalization and with a previously radiated arteriovenous malformation within the right primary visual cortex. Phase II monitoring demonstrated the pulvinar nucleus was not involved in subclinical seizures restricted to the primary visual cortex, but it did become involved in clinical events with more extensive seizure spread into higher visual cortical regions. She underwent responsive neurostimulation (RNS) with implantation of leads within the primary visual cortex. This case demonstrates the late propagation of epileptic activity from the visual cortex to the pulvinar nucleus and illustrates the pulvinar nucleus' connections with higher-order visual areas.

## Introduction

The thalamus is increasingly regarded as a useful target for circuit-based neurosurgical strategies to treat drug-resistant epilepsy (DRE). The growing understanding of the brain’s anatomical and functional connectivity has been valuable, to a great degree, in epilepsy and functional neurosurgery. The convergent-divergent nature of cortico-thalamo-cortical circuitry has engendered great interest in targeting the thalamus as a potential control point for neuromodulation, especially for patients who are otherwise considered high risk for surgical resection of an epileptogenic focus [1-5]. The anterior and centromedian nuclei of the thalamus, and, to a lesser degree, the pulvinar nucleus, have each been used as targets in this context [1,6,7].

In the following case report, we present an example of a patient with focal onset epilepsy of the occipital lobe, originating near a lesion within the primary visual cortex, with secondary generalization. This patient underwent phase II monitoring with stereotactic encephalography (SEEG) electrodes targeting the lesion and surrounding cortex as well as the pulvinar thalamus. Neurophysiological signals observed during subclinical and clinical seizure events demonstrated the selective involvement of the pulvinar thalamus in generalized seizures which originated in higher-order visual areas.

## Case presentation

**Figure 1:**
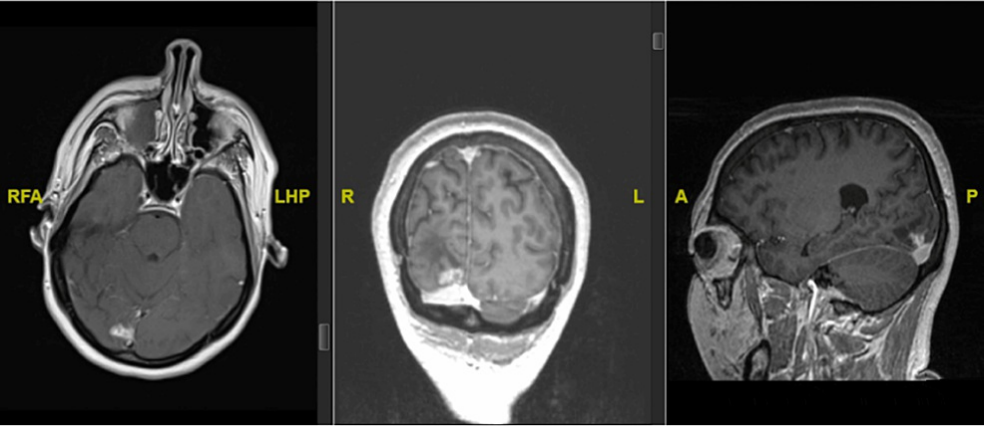
(From left to right) Axial, coronal, and sagittal views of MRI brain with contrast demonstrating a previously treated arteriovenous malformation in the right lingual gyrus with evidence of gliosis RFA, R: right. LHP, L: left. A: anterior. P: posterior LHP: left horizontal position

A 33-year-old female with a history of DRE presented with a medical history notable for a right occipital arteriovenous malformation (AVM) status post Gamma Knife radiosurgery at the age of 25. The AVM was abutting the calcarine sulcus in the primary visual cortex (Figure 1).

Her seizures started three months after Gamma Knife treatment. Semiology consisted of an initial flashing sensation in her left visual field, which progressed to formed images and then on occasion to secondary generalization with loss of consciousness, with no recollection of the antecedent events. She was trialed on multiple anti-seizure medications (ASMs) and eventually remained on two ASMs but with continuing seizures at the time of surgical evaluation. Her follow-up diagnostic cerebral angiogram revealed no residual flow through the AVM and the most recent magnetic resonance image (MRI) showed remnants of a right occipital lesion abutting the calcarine sulcus with post-treatment changes and surrounding enhancement. A preoperative neuro-ophthalmological evaluation revealed a partial left homonymous visual field defect involving the left inferior quadrant and left superior paracentral zone.

Scalp EEG captured one seizure lasting 16 minutes, which presented as a visualization of flashing lights, followed by unresponsiveness, and bilateral tonic-clonic activity, with forced head turn to the left and left arm extension. Ictal onset was over the right occipital region and evolved into rhythmic alpha activity and spike waves in the right hemisphere, which then generalized.

Based upon the patient’s semiology and phase 1 monitoring results, the epileptogenic zone (EZ) was agreed to be in the right occipital lobe where her prior AVM was located. Her surgical options included resection or ablation versus neuromodulation. Due to the eloquent location of her EZ, and the risk of additional vision loss or even complete homonymous hemianopsia with resective or ablative procedures, the patient elected to undergo responsive neurostimulation. Given a history of secondary generalization, and to optimize placement of the RNS electrodes, she was recommended to proceed first with phase II monitoring to characterize her seizure patterns and networks more precisely.

**Figure 2:**
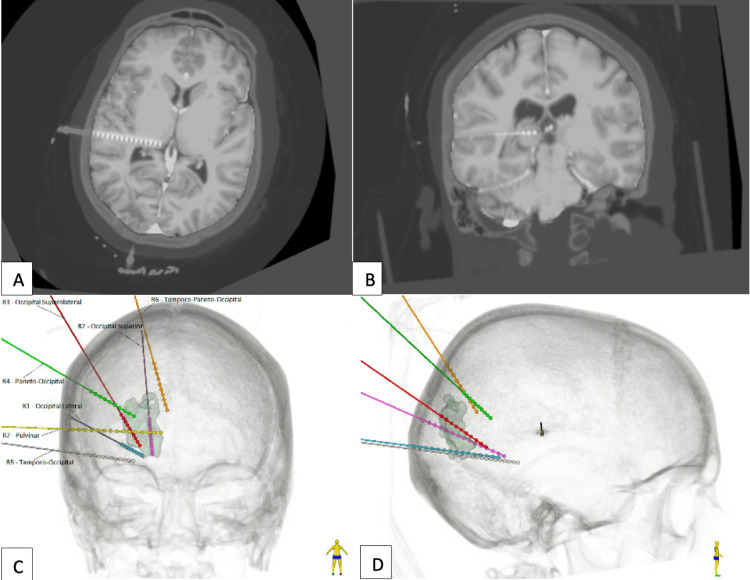
Axial (A) and coronal (B) views of MRI brain with a superimposed SEEG depth electrode implanted in the right pulvinar nucleus. Coronal (C) and (D) sagittal three-dimensional reconstruction of CT brain demonstrating implanted SEEG depth electrodes. SEEG: stereoelectroencephalography

The patient underwent CT-guided, robot-assisted placement of seven depth leads (with a total of 87 contacts) in the right hemisphere: an occipital lateral (OLAT), occipital superior (OSUP), occipital superolateral (OSLAT), parieto-occipital (POCC), temporo-occipital (TOCC), temporo-parieto-occipital (TPOCC), and pulvinar (PULV) leads (Figure 2).

The patient had five subclinical seizures on postoperative day (POD) one. Some events were restricted to the OLAT lead while others began in the OSUP lead before spreading to OLAT. The interictal activity involved primarily signals on those leads as well. No clinical seizures were associated with these events, which continued over POD two and three. These subclinical events were not associated with ictal signals in the pulvinar thalamus. ASMs were titrated down during this time to provoke clinical events.

**Figure 3:**
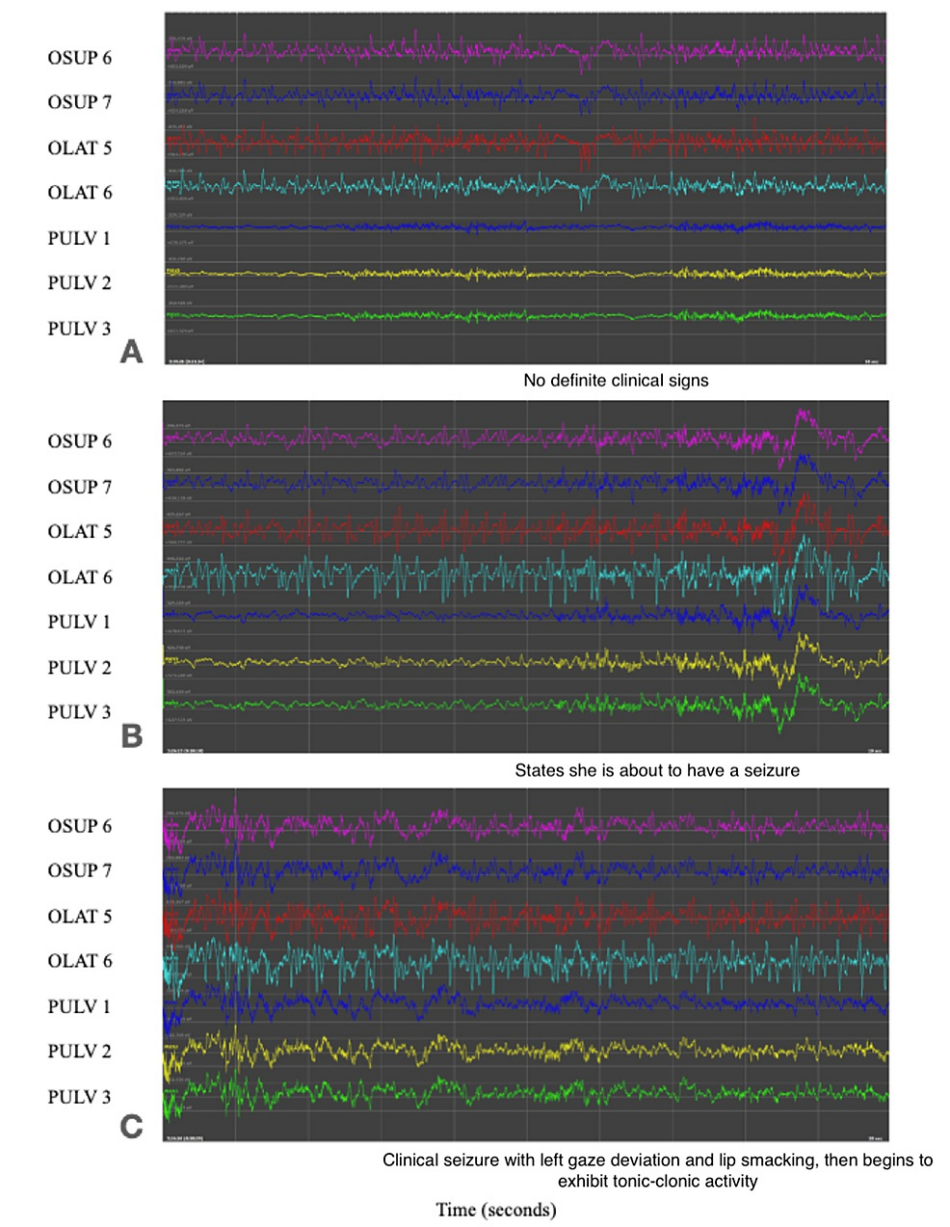
A-C. Electrographic recordings as seen in the OSUP contacts 6-7, OLAT contacts 5-6, and PULV contacts 1-3. Leads during a clinical seizure, with propagation of activity from the OSUP and OLAT leads in panel A, to the PULV lead approximately six minutes later in panels B and C OSUP: occipital superior; OLAT: occipital lateral; PULV: pulvinar

On POD three, the patient had a clinical seizure, which had an initial pattern of propagation as the subclinical seizures: These events started at one contact in the OSUP lead before involving a broader array of contacts in both OSUP and OLAT over the course of 17 minutes, particularly in the more distal contacts of these electrodes. Seizure activity ultimately propagated to involve the other, more anterior electrodes later during the episode, including pulvinar electrodes later in this recording, which culminated in a generalized tonic-clonic seizure (Figures 3A-3C). 

Over the following day, a total of 29 electrographic seizures were recorded with onset from either the OSUP or OLAT. This pattern of activity was consistent over the rest of the patient’s hospital stay. The depth electrodes were explanted on POD nine. The patient recovered well and was discharged the following day on her usual ASMs.

Based on this data, the OSUP and OLAT regions were implicated as early contributors to the patient’s clinical and subclinical events, with the pulvinar thalamus being involved only later in the clinical events with more extensive seizure spread. Two RNS implantation strategies were discussed: Two RNS depth leads could be implanted in the OLAT and OSUP trajectories where subclinical events were recorded and where clinical events began; or a single RNS lead could be implanted in V1 along either the OLAT or OSUP trajectories, with an additional lead in the pulvinar to address the broader spread of seizures during clinical events. After discussion, the comprehensive epilepsy team recommended the first strategy. The patient elected to proceed with this intervention and underwent an uncomplicated primary visual cortex RNS placement. She was discharged on POD one in stable condition. The patient presented eight weeks postoperatively to the emergency room for status epilepticus after missing three doses of one of her anti-epileptic drugs (AEDs). She was intubated for airway protection, admitted to the ICU, and the seizures were controlled. She was subsequently extubated, transferred to the floor level of care, and discharged in stable condition after her oxcarbazepine dose was increased.

**Figure 4:**
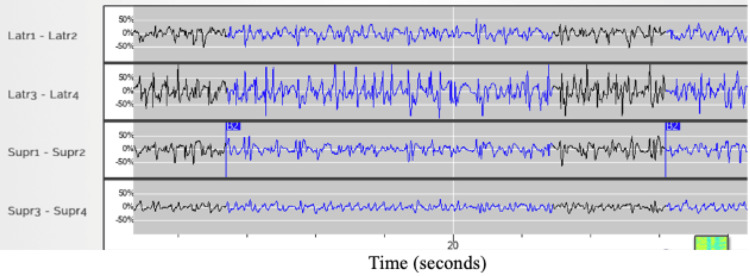
Long episode detection of high amplitude discharges in the superficial lateral contacts of the RNS system in the lateral occipital leads, labeled Latr3 - Latr4 Latr: lateral, Supr: superior, RNS: responsive neurostimulation

**Figure 5:**
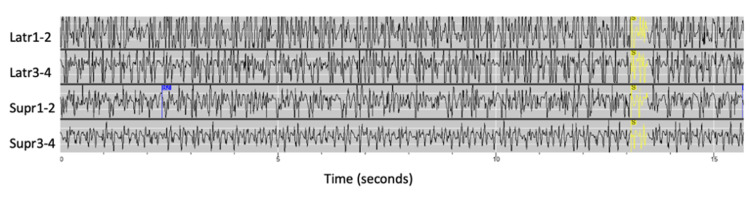
Fifteen-second ECOG recording as seen from the RNS system, labeled saturation, provoked by the detection of discharges (in yellow) with amplitudes above the threshold of detection of the lateral occipital RNS leads Latr: lateral, Supr: superior, RNS: responsive neurostimulation

The patient’s RNS data during this event, which was in detection mode, had detected three seizures on her day of presentation. The system detected high amplitude discharges reaching saturation thresholds in both leads after the detection of epileptiform discharges (Figures 4, 5).

Based on these findings and prior seizure recordings, stimulation settings were activated at the two-month follow-up appointment and set to be delivered in both leads for five burst stimulations with a current of 0.5 mA, frequency of 200 Hz, and a duration of 100 ms. At her most recent follow-up of six months post-op, the patient continues to do well with no stimulation side effects and reports a stable seizure frequency of one to two seizures/month up to this point. An ophthalmological evaluation postoperatively did not reveal any new visual field deficits.

## Discussion

The thalamus is increasingly considered a useful target to investigate both for the treatment of epilepsies that were previously considered “non-surgical,” such as primary generalized epilepsy and Lennox-Gastault and for seizures that involve eloquent regions that are not amenable to ablative or resective techniques. Because of the broad convergent-divergent nature of the cortico-thalamo-cortical system, the thalamus can be a target for neuromodulation to palliate debilitating seizures, including in patients with impairment of consciousness where cortico-thalamic circuits are implicated [8,9].

Patients with DRE of posterior quadrant origin traditionally present challenges for surgical intervention due to the risk of visual loss with resective approaches. Prior studies of open- and closed-loop systems targeting the anterior nucleus of the thalamus (ANT) included patients with this pathology in a very small subset of their subjects, and, as a result, it is difficult to draw conclusions about the efficacy of thalamic neuromodulation of the ANT for these patients [5,10,11].

Considering other thalamic nuclei, the pulvinar nucleus is of interest due to its connections with the visual pathways. Associative thalamic nuclei, such as the pulvinar facilitate cortico-cortical communication via an indirect, transthalamic pathway (cortico-thalamo-cortical), rendering them potentially valuable targets for the modulation of cortico-cortical seizure spread. Primate studies describe pulvinar connections to V1, V2, and V4 in the visual cortex, in addition to the higher visual areas in the inferior temporal cortex [12-14]. In our patient, epileptic activity recorded in the PULV lead later in the seizure episode prior to generalization provides further evidence of the involvement of the pulvinar nucleus in epileptic networks, especially in those that involve the visual pathways.

Investigations using iEEG of the pulvinar nucleus in patients with posterior quadrant epilepsy showed its involvement simultaneously or early during seizure propagation from the visual cortex [1,7]. In a case series of three patients with posterior quadrant onset epilepsy, RNS targeting the pulvinar nucleus resulted in a greater than 50% reduction in their seizures. Two of these patients had depth electrodes implanted within the pulvinar nucleus during phase II monitoring, which showed either simultaneous or early involvement of the pulvinar nucleus [7]. In our case, due to the consistent evidence of seizure onset from the occipital superior and lateral regions, and that the majority of her episodes were subclinical seizures of those regions, RNS leads were implanted in these two regions to further collect evidence and abort seizures at their onset. Late involvement of the pulvinar nucleus was considered a result of the propagation of uncontrolled seizures along higher-order visual pathways, which is the rationale behind the deferral of thalamic RNS in this case.

## Conclusions

This case demonstrates the late propagation of epileptic activity from the visual cortex to the pulvinar nucleus before immediately culminating in a generalized tonic-clonic seizure with impairment of consciousness. This is in line with prior evidence of this nucleus’ connection with higher-order visual areas as seen in various case reports and animal studies. Recruitment of patients with this challenging pathology in future trials is essential to tailor thalamic neuromodulation solutions for a significant number of patients.
